# Neuroprotective effects of intrastriatal injection of rapamycin in a mouse model of excitotoxicity induced by quinolinic acid

**DOI:** 10.1186/s12974-017-0793-x

**Published:** 2017-01-31

**Authors:** Soraya Wilke Saliba, Erica Leandro Marciano Vieira, Rebeca Priscila de Melo Santos, Eduardo Candelario-Jalil, Bernd L. Fiebich, Luciene Bruno Vieira, Antonio Lucio Teixeira, Antonio Carlos Pinheiro de Oliveira

**Affiliations:** 1 Department of Pharmacology, Universidade Federal de Minas Gerais, Avenida Antonio Carlos, 6627, 31270-901 Belo Horizonte, MG Brazil; 2 Department of Internal Medicine, Universidade Federal de Minas Gerais, Belo Horizonte, MG Brazil; 3 Department of Neuroscience, University of Florida, Gainesville, FL 32610 USA; 4 Department of Psychiatry, University of Freiburg Medical School, Hauptstr. 5, 79104 Freiburg, Germany

**Keywords:** Neurodegeneration, mTOR, Rapamycin, Quinolinic acid, Inflammation, Glutamate, Neurotrophic factors

## Abstract

**Background:**

The mammalian target of rapamycin (mTOR) is a kinase involved in a variety of physiological and pathological functions. However, the exact role of mTOR in excitotoxicity is poorly understood. Here, we investigated the effects of mTOR inhibition with rapamycin against neurodegeneration, and motor impairment, as well as inflammatory profile caused by an excitotoxic stimulus.

**Methods:**

A single and unilateral striatal injection of quinolinic acid (QA) was used to induce excitotoxicity in mice. Rapamycin (250 nL of 0.2, 2, or 20 μM; intrastriatal route) was administered 15 min before QA injection. Forty-eight hours after QA administration, rotarod test was performed to evaluate motor coordination and balance. Fluoro-Jade C, Iba-1, and GFAP staining were used to evaluate neuronal cell death, microglia morphology, and astrocytes density, respectively, at this time point. Levels of cytokines and neurotrophic factors were measured by ELISA and Cytometric Bead Array 8 h after QA injection. Striatal synaptosomes were used to evaluate the release of glutamate.

**Results:**

We first demonstrated that rapamycin prevented the motor impairment induced by QA. Moreover, mTOR inhibition also reduced the neurodegeneration and the production of interleukin (IL)-1β, IL-6, and tumor necrosis factor (TNF)-α induced by excitotoxic stimulus. The lowest dose of rapamycin also increased the production of IL-10 and prevented the reduction of astrocyte density induced by QA. By using an in vitro approach, we demonstrated that rapamycin differently alters the release of glutamate from striatal synaptosomes induced by QA, reducing or enhancing the release of this neurotransmitter at low or high concentrations, respectively.

**Conclusion:**

Taken together, these data demonstrated a protective effect of rapamycin against an excitotoxic stimulus. Therefore, this study provides new evidence of the detrimental role of mTOR in neurodegeneration, which might represent an important target for the treatment of neurodegenerative diseases.

**Electronic supplementary material:**

The online version of this article (doi:10.1186/s12974-017-0793-x) contains supplementary material, which is available to authorized users.

## Background

Huntington’s disease (HD) is an autosomal dominant inherited neurodegenerative disease, which manifests with motor dysfunction, psychiatric symptoms, and cognitive decline. HD is characterized predominantly by neurodegeneration in the striatum and cortex, as well as in the thalamus and cerebellum [1, 2]. Injection of quinolinic acid (QA) into the striatum of rodents is used as a neurotoxic animal model of HD since it mimics the deficits seen in early stages of neurodegeneration in this disease, leading to the loss medium spiny projection neurons (MSNs) in the striatum [3–5].

QA is an agonist of the NMDA receptor [6]. High concentrations of this compound are shown to increase glutamate release and also the influx of calcium ions leading to the excitotoxic process. Besides, it inhibits the glutamate reuptake by astrocytes [6–10]. In addition to excitotoxicity, QA also induces mitochondrial dysfunction [11] and inflammation [5].

The phosphatidylinositol 3-kinase/mammalian target of rapamycin (PI3K/mTOR) pathway is involved in many physiological and pathological processes. The activation of this pathway by different stimuli induces the phosphorylation of phosphatidylinositol 4,5-bisphosphate (PIP_2_) by PI3K enzyme to generate phosphatidylinositol-3,4,5-trisphosphate (PIP_3_). PIP_3_ facilitates the phosphorylation of Akt by the phosphoinositide-dependent kinase-1 (PDK-1) and mTOR complex 2 (mTORC2). The phosphorylation of the Akt leads to further catalytic activity changes of downstream targets, such as forkhead-related transcription factor 1, Bad, glycogen synthase kinase-3 (GSK-3), and mTOR complex 1 (mTORC1) [12, 13].

mTOR is a serine/threonine protein kinase that participates in the regulation of cell growth and proliferation, as well as in several intracellular processes, such as mRNA transcription and translation, autophagy and cytoskeletal organization. mTOR is involved in the pathophysiology of different conditions, being, for example, a potential pharmacological target for the treatment of cancer and diabetes [14]. mTOR forms two distinct functional complexes termed mTOR complexes 1 (mTORC1) and 2 (mTORC2). The mTORC1 is the primary target of inhibition by the drug rapamycin, but higher concentration of rapamycin or chronic treatment can also interfere on mTORC2 regulation [15]. In this context, the implication of mTOR in neuropathological conditions has being suggested. In fly and mouse genetic models of HD, inhibition of mTOR by rapamycin induced autophagy and reduced toxicity of polyglutamine expansions [16]. Administration of rapamycin reduced the number of activated microglia/macrophages and increased the number of surviving neurons at the cortical region near the site of the lesion in an animal model of head injury [17]. Moreover, this kinase regulates the expression of type 1 glutamate transporter in astrocytes [18].

The effects of mTOR inhibition on the inflammatory process are not clear. mTOR inhibition in primary microglial cultures increased the production of cyclooxygenase (COX)-2 and microsomal prostaglandin E synthase-1 (mPGES-1) after stimulation with LPS and poly(I:C) [19, 20]. In peripheral blood mononuclear cells, rapamycin increased the release of interleukin (IL)-12, a pro-inflammatory cytokine, and reduced IL-10 after LPS stimulation [21]. On the other hand, in microglia cultures stimulated with LPS, rapamycin reduced the expression of nitric oxide synthase (NOS) and COX-2 [22, 23]. Thus, this kinase could be involved in the excitotoxicity and neuroinflammation observed in neurodegenerative diseases.

Although few studies have evaluated the role of mTOR in traumatic brain injury and genetic models of neurodegeneration, its role in the pathological processes associated with excitotoxic injury, an event present in neurodegenerative diseases, is poorly understood. Therefore, in the present study, we investigated the effect of mTOR inhibition on neurodegeneration and neuroinflammation induced by intrastriatal injection of QA.

## Methods

### Animals

Male C57Bl/6 mice (10–12 weeks), bred in the vivarium of the Federal University of Minas Gerais, were used. Animals were kept under standard laboratory conditions, maintained on 12-h light/dark cycle with temperature of 24 °C and free access to water and food. Mice were acclimatized in the experimental room 30 min before the test. All the experiments were performed between 8 a.m. and 5 p.m. All the experiments were approved by the Ethics Committee on Animal Experimentation (CEUA) of the Federal University of Minas Gerais (protocol number 327/2012).

### Drugs

Rapamycin (LC Laboratories, Woburn, MA, USA), a mTOR inhibitor, was first dissolved in DMSO 100% (Sigma-Aldrich, St Louis, MO, USA) to create a 10 mM stock solution. Thereafter, the drug was diluted with saline to reach the concentrations of 0.2, 2, or 20 μM. Two hundred fifty nanoliters of rapamycin were administered into the right striatum. Therefore, the total amount of rapamycin injected were doses of 5 × 10^−5^, 5 × 10^−4^, or 5 × 10^−3^ nmol. The vehicle had the same concentration of DMSO that the rapamycin solution. Quinolinic acid (Sigma-Aldrich, St Louis, MO, USA) was dissolved in phosphate-buffered saline (PBS, pH 7.0) and administered unilaterally in the right striatum (250 nL of a 0.8 M solution). Thus, the total amount of QA injected was 200 nmol.

### Intrastriatal administration of rapamycin and QA

In order to avoid peripheral pharmacological responses and to investigate the local actions of mTOR inhibition, we injected rapamycin directly in the striatum. Animals were anesthetized with a solution of ketamine and xylazine (80 and 8 mg/kg, respectively) to implant the cannula guide unilaterally into the right striatum. The following coordinates were used for the administration of rapamycin and QA in the striatum: anteroposterior +0.6 mm, mediolateral −2.1 mm, and dorsoventral −2.2 mm from the bregma based on the atlas by Paxinos and Watson [24] (Additional file 1: Figure S1). After 7 days, rapamycin and QA were injected using a 30-gauge stainless steel needle attached to a Hamilton syringe. Rapamycin or vehicle (5% DMSO in saline) was injected at 0.08 μL/min, and the injection needle was left in place for another 1 min to allow diffusion and to avoid the reflux of the solution. After 15 min, QA or PBS were administered over a period of 3 min, and the injection needle was left in place for another 1 min to allow diffusion and to avoid the reflux of the solution.

Animals were divided into six groups: vehicle + PBS; vehicle + QA; rapamycin 0.2 μM + PBS; rapamycin 0.2 μM + QA, rapamycin 2 μM + QA, and rapamycin 20 μM + QA. The behavioral and biochemical assessments were performed as shown in Additional file 1: Figure S1.

### Body weight

Body weight of the animals was evaluated before the administration of the drugs (QA and/or rapamycin) and on the last day of the study.

### Rotarod activity

Motor coordination and balance were assessed using rotarod (Insight®, São Paulo, Brazil). Before the surgery for implantation of the guide cannula, all the animals were given a prior training session on the rotarod apparatus, which consisted of three consecutive days at low rotational speeds (14, 17, 2,0 and 24 rpm) for 3 min each. Seven days after the surgery, mice were placed on the rod starting at 14 rpm and accelerating to 37 rpm within 5 min [25]. Three trials were conducted, and the time that the animals stayed on the rod before falling off was measured and considered the baseline performance. After 2 days of QA or PBS administration, the latency for the animals to fall off was recorded again for each animal.

### Brain extraction and sectioning

Two days after the administration of QA or PBS, mice were anesthetized with a solution of ketamine and xylazine (80 and 8 mg/kg, respectively), and perfused with PBS 1× followed by 4% paraformaldehyde (PFA). The brains were harvested, and then fixed with the same solution for 24 h at 4 °C, and cryoprotected for additional 2 days by immersion in 30% sucrose at 4 °C. The striatum was sectioned at 30 μm with a cryostat (Leica Biosystem, USA) at −20 °C. A series of four coronal sections of the central striatum was mounted for immunofluorescence analysis and stained with Fluoro-Jade C (FJC), ionized calcium-binding adapter molecule 1 (Iba-1), and glial fibrillary acidic protein (GFAP).

### Assessment of degenerating neurons

FJC analysis was used to quantify the number of degenerating neurons in the striatum. Brain sections were mounted on gelatin-coated slides, air-dried, and subjected to FJC staining. Slides were first immersed in a solution containing 1% NaOH in 80% ethanol for 5 min. They were rinsed for 2 min in 70% ethanol and for 2 min in distilled water, and then incubated in 0.06% potassium permanganate solution for 20 min. Thereafter, the slides were rinsed with water for 2 min and incubated in 0.0001% FJC solution for 20 min. The 0.0001% FJC solution was prepared by first making a 0.01% stock solution of FJC dye in distilled water and then adding 1 mL of the stock solution to 99 mL of 0.1% acetic acid. Slides were washed two times each for 1 min and then air-dried on a slide warmer at 37 °C. After 10 min, sections were washed, dried, cleared in xylene, and cover slipped in DPX mounting media. Sections were imaged in a Zeiss fluorescent microscope (Zeiss, Oberkochen, Germany) using a 488 nm excitation. Quantification of fluorescence intensity was done using ImageJ software. The number of fluorescent points in each picture, indicating the dead neurons, was counted by an investigator blinded to the experimental groups.

### Assessment of glial cells

Evaluation of glial cells, such as microglia and astrocytes, was determined by performing Iba-1 and GFAP immunofluorescence, respectively. In brief, central striatum sections (30 μm) were first blocked with 4% bovine serum albumin (BSA) containing 0.05% Triton in Tris-buffered saline (TBS) and then incubated with the indicated primary antibodies (1:400 with anti-Iba1 from WAKO or 1:1000 with GFAP from Santa Cruz Biotechnology) overnight at 4 °C. Slides were then incubated with secondary antibody for 1 h at room temperature. Rabbit highly cross-adsorbed AlexaFluor 594 or AlexaFluor 488 secondary antibody (Invitrogen, Carlsbad, CA, USA) was used to detect Iba-1 or GFAP, respectively. Sections were imaged in a Zeiss fluorescent microscope (Zeiss, Oberkochen, Germany) and quantified with ImageJ software. The cell area of microglia and astrocyte density were estimated using ImageJ software, and the results presented as cell area divided by the number of cells for microglia and for astrocytes, intensity of the signal divided by the area.

### Quantification of neurotrophic factors and cytokines in striatum

Eight hours after the administration of QA or PBS, the right and left striatum were dissected and kept at −80 °C until tested. Samples were homogenized in 200 μL of protease inhibitor solution [NaCl 0,4 M; Tween 20 (Synth) 0.05%; bovine serum albumin (BSA) (Inlab Confiança) 0.5%; phenylmethylsulfonylfluoride (Calbiochem) 0.1 mM; benzethonium chloride (Vetec) 0.1 mM; EDTA 10 mM; 20 UI de aprotinin (Sigma), diluted in PBS]. Homogenates were centrifuged at 4 °C at 10,000 rpm, aliquoted, and stored at −80 °C until analysis. Brain-derived neurotrophic factor (BDNF), nerve growth factor (NGF), and interleukin (IL)1β protein levels were assessed using ELISA kits (R&D Systems, Abingdon, UK) according to the manufacturer protocol. IL-2, IL-4, IL-6, interferon (IFN) γ, tumor necrosis factor alpha (TNF-α), IL-10, and IL-17A were assessed using Th1/Th2 kit (BD Pharmigen, CA, USA) by Cytometric Bead Array (CBA) method according to the protocol of the manufacturer. The results of the ELISA and CBA were expressed as pg/mg of total protein.

### Preparation of synaptosomes

Male C57Bl/6 mice (10–12 weeks) were decapitated, and the striatum was removed and homogenized in 1:10 (*w*/*v*) 0.32 M sucrose containing 0.25 mM dithiothreitol and 1 mM EDTA. Homogenates were then submitted to low-speed centrifugation (1000×*g*/10 min), and the isolated nerve terminals (synaptosomes) were purified from the supernatant by discontinuous Percoll density gradient centrifugation [26]. The isolated synaptosomes were resuspended in 400 μL Krebs-Ringer-HEPES (KRH) solution (124 mM NaCl, 4 mM KCl, 1.2 mM MgSO_4_, 10 mM glucose, 25 mM HEPES, pH 7.4) without addition of CaCl_2_ and were kept on ice until use for the quantification of glutamate release.

### Measurement of continuous glutamate release

Glutamate release was measured by continuous fluorometric assay as described by Nicholls et al. [27]. Briefly, 300 μL of synaptosomes with CaCl_2_ (1 mM), NADP^+^ (1 mM), and KRH medium was added in wells of the ELISA plate. First, to evaluate the effect of QA on glutamate release, glutamate dehydrogenase (GDH) (35 units/well) was mixed with the striatal synaptosomes followed by the addition of QA (0.1, 0.5, 5, and 10 mM) or PBS or KCl 33 mM. To study the effect of rapamycin on glutamate release, synaptosomes were previously incubated with rapamycin (10^−4^, 10^−3^, 0.05, or 0.25 nM) for 15 min and GDH (35 units/well) were added to synaptosomes. Thereafter, QA 5 mM or PBS or KCl 33 mM were added. The glutamate measurement was performed by the increase in fluorescence due to the production of NADPH^+^ in the presence of glutamate dehydrogenase and NADP^+^. Calibration curves were prepared in parallel by adding known amounts of glutamate to the reaction medium. Fluorescence emission was recorded using a fluorometer (Biotek Synergy 2) at 450 nm, and the excitation wavelength was set at 360 nm. Results are expressed as percentage of the QA group (mean ± SEM).

### Statistical analysis

Data are presented as mean ± SEM. The data was analyzed using one-way analysis of variance (ANOVA) followed by Newman-Keuls post-test and two-way ANOVA followed by Bonferroni post-tests as appropriate. The level of statistical significance was set at a *p* value less than 0.05. Graph Pad Prism (Graph Pad Software, San Diego, CA) was used for performing all statistical analysis.

## Results

### Rapamycin prevents motor impairment induced by QA

**Fig. 1:**
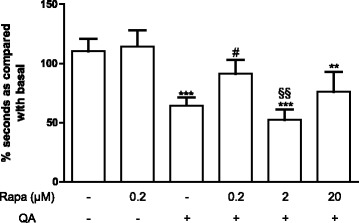
Effect of rapamycin on rotarod activity 2 days after QA administration (*n* = 8–15 animals/group). Rapamycin was injected 15 min before QA injection into the striatum and after 2 days, rotarod performance was evaluated. Results are expressed as mean ± SEM (% of basal performance). ***p < 0.001 and **p < 0.01 as compared to vehicle + PBS. ^#^ *p* < 0.05 as compared to vehicle + QA. ^§§^ *p* < 0.01 as compared to Rapa 0.2 + QA (two-way ANOVA followed by Bonferroni test)

QA intrastriatal injection leads to a significant decrease in motor coordination as observed in HD animal models [28]. To test whether rapamycin prevents motor dysfunction induced by QA, animals were evaluated by rotarod activity. Intrastriatal QA administration significantly reduced rotarod performance (% of fall off time) as compared to control animals (Fig. 1). The lower dose of rapamycin (0.2 μM/250 nl) prevented the motor impairment induced by QA. This same dose of rapamycin did not alter the motor performance of the animals treated with PBS. The other concentrations of rapamycin did not alter the performance of the animals in the rotarod in comparison with the QA-injected group (Fig. 1). None of the treatments produced any significant change in body weight (Additional file 2: Figure S2).

### Rapamycin decreases neurodegeneration of striatal neurons induced by QA

**Fig. 2:**
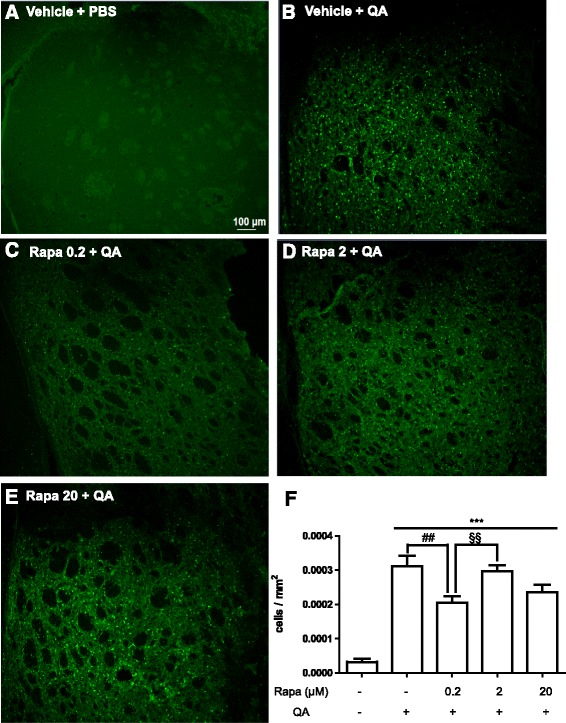
Effect of rapamycin on neurodegeneration 2 days after QA administration (*n* = 5–7 animals/group). Rapamycin was injected 15 min before QA injection into the striatum and after 2 days, and dead neurons were evaluated. **a**–**e** Representative FJC of the right striatum in 10×. **f** A graph of the FJC data of the right striatum. Results are expressed as mean ± SEM. ***p < 0.001 as compared to vehicle + PBS. ^##^ *p* < 0.001 as compared to vehicle + QA. ^§§^ *p* < 0.01 as compared to Rapa 0.2 + PBS (one-way ANOVA followed by Newman-Keuls test)

Neostriatal atrophy is the morphologic hallmark of HD [29]. In order to investigate whether the effect of rapamycin on the motor impairment is due to a reduced neuronal cell death, we performed Fluoro-Jade C staining. QA significantly increased the neuronal cell death in the striatum as compared with negative control (Fig. 2a, b). It is possible to observe a change in tissue morphology in the ipsilateral striatum in all groups with QA administration, suggesting tissue degeneration (Fig. 2b-e). Importantly, rapamycin at the dose of 0.2 μM/250 nl decreased neuronal cell death induced by QA (Fig. 2c and f). Although a trend towards a decrease in QA-induced damage is also observed with the higher dose of rapamycin, no statistical difference was observed (Fig. 2e and f).

### Rapamycin alters glutamate release induced by QA in synaptosomes

**Fig. 3:**
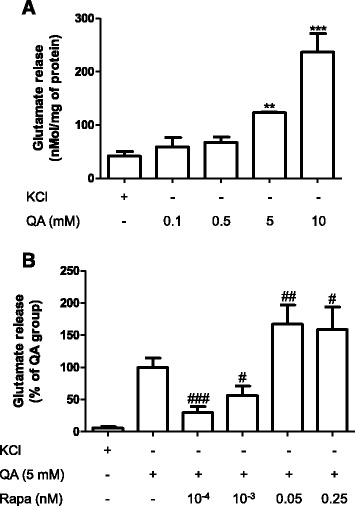
Effect of different concentrations of QA and rapamycin on the glutamate release in striatal synaptosomes. **a** Synaptosomes were incubated with different concentration of QA (0.1, 0.5, 5, and 10 mM) (*n* = 2–3). **b** Previously, synaptosomes were incubated with rapamycin (10^−4^, 10^−3^, 0.05, or 0.25 nM) for 15 min, and after, QA 5 mM were added. The glutamate release was evaluated by continuous fluorometric assay. Results are expressed as mean ± SEM (*n* = 3–10 independent experiments/group). **p < 0.05, ***p < 0.01 as compared with KCl 33 mM. ^###^ *p* < 0.001, ^##^ *p* < 0.01, and ^#^ *p* < 0.05 as compared with QA 5 mM (one-way ANOVA followed by Newman-Keuls test)

In order to investigate whether rapamycin would modulate neurotransmitter release, an effect that could contribute to the neuroprotective effect of rapamycin, we evaluated its effect on glutamate release in synaptosomes. We first demonstrated that QA increases the release of glutamate in synaptosomes prepared from mice striatum (Fig. 3a). Rapamycin reduced QA-induced glutamate release from synaptosomes at the lower concentrations (0.0001–0.001 nM) and enhanced the release of this neurotransmitter at higher concentrations (>0.05 nM, Fig. 3b).

### Rapamycin does not alter neurotrophic factors production in the striatum after QA administration

**Fig. 4:**
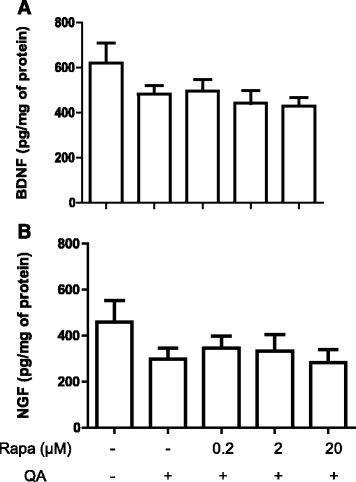
Effect of rapamycin on the levels of neurotrophic factors in the striatum after 8 h of QA administration. Rapamycin was injected 15 min before QA injection into the striatum, and the quantification of BDNF (*n* = 6–7 animals/group) (**a**) and NGF (*n* = 5–7 animals/group) (**b**) was determined after 8 h of the QA injection using ELISA. Results are expressed as mean ± SEM. There are no statistically significant differences among treatment groups (one-way ANOVA followed by Newman-Keuls test)

We next evaluated whether rapamycin would alter the production of neurotrophic factors shortly after the injection of the excitotoxic stimulus, which could modify the neuronal cell death 2 days after injection. QA did not statistically alter the production of BDNF and NGF 8 h after the injection, although a trend towards reduction was observed. Moreover, rapamycin also did not affect the levels of these neurotrophins at this time point (p_BDNF_ = 0.1688 and p_NGF_ = 0.4336) (Fig. 4).

### Rapamycin alters cytokine production in the striatum after QA administration

**Fig. 5:**
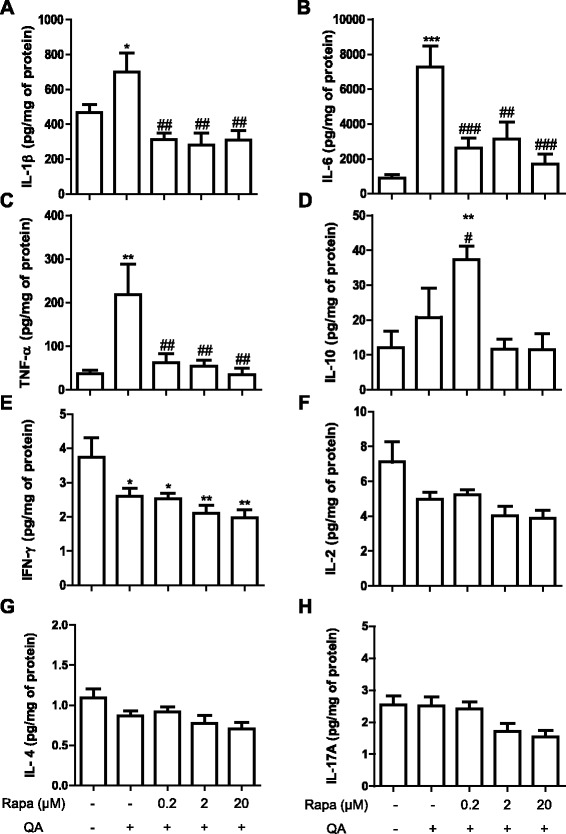
Effect of rapamycin on IL-1β (**a**), IL-6 (**b**), TNFα (**c**), IL-10 (**d**), IFN-γ (**e**), IL-2 (**f**), IL-4 (**g**), and IL-17A (**h**) levels in the striatum after 8 h of QA administration (*n* = 5–7 animals/group). Results are expressed as mean ± SEM. ***p < 0.001, **p < 0.01, and *p < 0.05 as compared to vehicle + PBS. ^###^ *p* < 0.001, ^##^ *p* < 0.01, and ^#^ *p* < 0.05 as compared to vehicle + QA (one-way ANOVA followed by Newman-Keuls test)

In order to investigate whether rapamycin could alter the production of inflammatory mediators, which could be a consequence of the neurodegenerative process and also contribute to neuronal damage, we investigated the production of cytokines 8 h after the stimulus. Quantification of cytokines was performed in the ipsilateral hemisphere. As shown in Fig. 5a–c, IL-1β, IL-6, and TNFα were increased by QA injection. All the concentrations of rapamycin reduced these cytokines induced by QA (Fig. 5a–c). On the other hand, although a trend towards increase in IL-10 was induced by QA (Fig. 5d), no statistical difference was observed. However, the lower dose of rapamycin (0.2 μM/250 nl) increased the levels of this cytokine in comparison with QA group (Fig. 5d).

QA also reduced the levels of IFNγ (Fig. 5e), which was not significantly altered by rapamycin treatment. None of the other cytokines (i.e., IL-2, IL-4, IL-17A) were altered by the treatment with QA and/or rapamycin at this time point (Fig. 5f–h).

### Rapamycin does not alter the morphology of microglia in the striatum of QA-treated animals

**Fig. 6:**
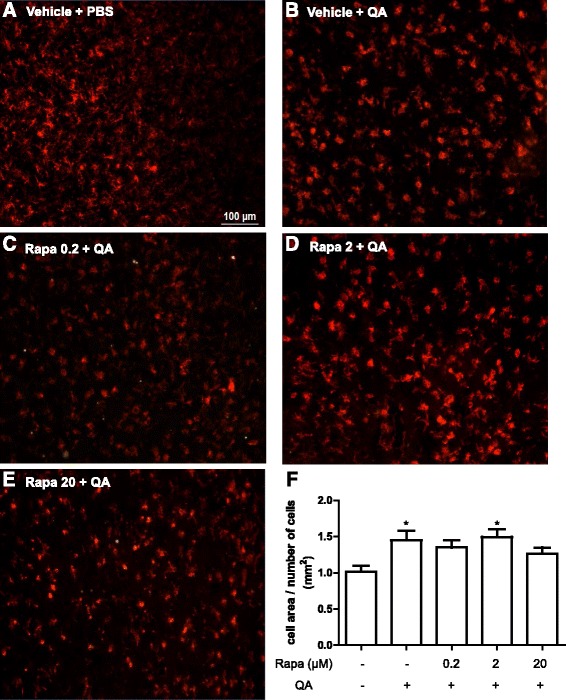
Effect of rapamycin on microglia activation after 2 days of QA administration (*n* = 3–4 animals/group). Rapamycin was injected 15 min before QA injection into the striatum, and after 2 days, Iba-1 staining was performed. **a**–**e** Representative images of the staining with anti-Iba-1 antibody in the right striatum at 20× (ipsilateral). **f** Quantitative analysis of the cell area divided by the number of the cells. Results are expressed as mean ± SEM. *p < 0.05 as compared with vehicle + PBS (one-way ANOVA followed by Newman-Keuls test)

We next evaluated whether the alteration in production of inflammatory mediators would be accompanied by changes in microglial morphology. We evaluated microglia at 48 h after the stimulus because the activation of these cells would be present due to severe neurodegenerative process at this time point. Microglia activation, as revealed by their retraction of their projections and enlarged cell bodies, was increased in the QA group (Fig. 6b). We performed an analysis to investigate whether the cell size would be affected by rapamycin. For this, we determined the area of the cells and divided this area by the number of cells. We observed that the cell size increased with QA injection, but no difference was observed with the groups treated with rapamycin (Fig. 6c-f).

### Rapamycin prevented the reduction in astrocyte density in the striatum of QA-treated animals

**Fig. 7:**
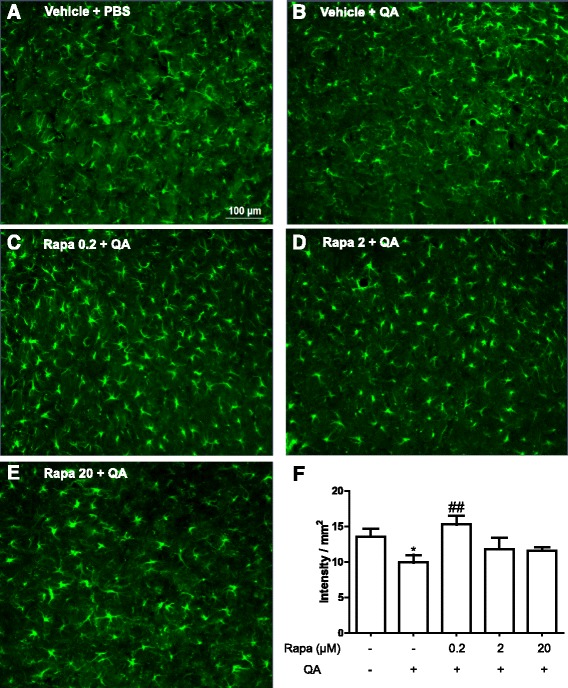
Effect of rapamycin on astrocyte density after 2 days of QA administration (*n* = 3–4 animals/group). Rapamycin was injected 15 min before QA injection into the striatum, and after 2 days, GFAP staining was performed. **a**–**e** Representative images of the staining with GFAP antibody in the right striatum at 20× (ipsilateral). **f** Quantitative analysis of the density divided by the area. Results are expressed as mean ± SEM. *p < 0.05 as compared with vehicle + PBS and ^##^ *p* < 0.01 as compared to vehicle + QA (one-way ANOVA, Newman-Keuls test)

Astrocytes possess various physiological functions and also contribute to inflammation associated with neurodegeneration. Therefore, we evaluated whether these cells were affected by QA and the effect of rapamycin on it. As demonstrated in Fig. 7, QA reduced the astrocyte density, which was prevented by the treatment with the lower dose of rapamycin (0.2 μM/250 nl), but not by the others.

## Discussion

In the present study, we evaluated the effect of rapamycin in the excitotoxicity induced by intrastriatal injection of QA. We demonstrated that this drug prevented the motor impairment and the reduction of astrocyte density, reduced neuronal cell death, and attenuated the inflammatory process associated with neurodegeneration induced by QA.

We first demonstrated that the lower, but not the higher doses of rapamycin, prevented the motor impairment induced by QA. Although it is difficult to explain this effect with the results of the present work, drugs may have variable effects depending on the concentration. Some studies have shown that higher concentrations of rapamycin inhibit not only mTORC1 but also mTORC2, as well as influence calcium signaling [15, 30]. Low concentrations of rapamycin can improve learning and memory [31, 32]. However, high concentrations of rapamycin completely inhibit mTOR, affect long-term memory facilitation and consolidation, and cause amnesia [32, 33].

In order to investigate whether the effect of rapamycin on the motor impairment was due to a reduced neuronal cell death, we performed Fluoro-Jade C analysis. We showed that the lower dose of rapamycin reduced the neuronal death induced by the excitotoxic stimulus. This neuroprotection induced by rapamycin could be due to reduced neuronal firing. Indeed, rapamycin has been shown to reduce excitatory postsynaptic current (EPSC) in glutamatergic neurons [34]. Moreover, rapamycin decreased the neurotoxic effect of glutamate insult on hippocampal neuronal cultures through a mechanism involving increased autophagy [35], and also, in a rat model of NMDA-induced retinal injury [36, 37] and kainate rat model [38], rapamycin has been shown to confer neuroprotection against neurotoxicity. This neuroprotective effect of rapamycin have also been demonstrated in other neurodegenerative models, such as in traumatic brain injury model [39, 40], mouse model of early-stage Alzheimer’s disease (AD)-type tauopathy [41] and spinal cord injury [42]. On the other hand, our group has recently demonstrated that rapamycin does not alter the neuronal cell death induced by intrahippocampal injection of amyloid beta [43]. The effects of rapamycin seem to be dependent on the type or period of stimuli and dose [44].

Tavares et al. [9] showed that QA also enhanced the release of glutamate in rat synaptosomes. In the present study, we demonstrated that QA increased the release of glutamate from mice striatal synaptosomes. Interestingly, rapamycin reduced QA-induced glutamate release at low concentrations and produced the opposite effect at higher concentrations. Although it might be speculative to extrapolate the effect of specific concentrations used in vitro to doses used in in vivo experiments, these results suggest that low concentrations of rapamycin, by decreasing the release of glutamate, would lead to a reduced neuronal death, consequently avoiding the motor impairment induced by QA. In accordance with our study, hyperactivation of mTOR increased the evoked synaptic responses in glutamatergic neurons, and rapamycin reduced this response [34]. Therefore, although the main role of mTOR is regarded as pro-survival, inhibition of this kinase might be neuroprotective in excitotoxic conditions.

Neurotrophic factors such as BDNF and NGF promote neuronal survival as well as cell proliferation and differentiation of neurons [45]. In the striatum, BDNF is important for neuronal activity and survival [46]. Mishra and Kumar [47] have shown that a single intrastriatal bilateral QA administration in Wistar rats decreased BDNF after 21 days. In the current study, no change was observed in the levels of BDNF and NGF after injection of QA. The short time point, i.e., 8 h after QA, at which we measured these neurotrophins could possibly explain this discrepancy between the studies. Furthermore, it is also possible that these neurotrophic factors may not contribute to the neuroprotective effect of rapamycin in this model.

The production of cytokines usually occurs immediately after excitotoxic events [48]. Schiefer et al. [48] have shown that the unilateral administration of 240 nmol of QA in the striatum of Wistar rats increased IL-6 levels after 3 h, and the maximal levels were observed at 6 h, followed by a reduction at 24 h. Therefore, we used 8 h after stimulation with QA to evaluate the levels of the cytokines. We observed that QA-injected mice increased pro-inflammatory cytokines when compared with all rapamycin-treated groups and PBS-injected controls. In our model, all concentrations of rapamycin reduced the content of IL-1β, IL-6, and TNFα induced by QA. Indeed, other studies have shown that rapamycin inhibits the production of IL-1β and IL-6 in LPS-stimulated microglia [49] and reduces and delays the LPS increase in IL-1β and TNF-α in a rat model of absence epilepsy [50]. On the other hand, we have shown that only the low dose of rapamycin enhanced IL-10, an anti-inflammatory cytokine that has been demonstrated to exhibit neuroprotective effects [51, 52]. Although the most important source of cytokines is microglia, it has been shown that QA also induced the production of IL-1β by human astrocytes [53]. Furthermore, rapamycin reduced the release of IL-1β and TNF in astrocytes exposed to ischemia [54]. Thus, it is possible that, in our model, rapamycin reduced the production of cytokines induced by QA also in astrocytes. As demonstrated previously, in addition to excitotoxicity, neuronal cell death can also occur due to an enhanced inflammatory process [55]. However, in the current study, a sole reduction in the production of pro-inflammatory mediators does not seem to influence the neuronal death as all doses of rapamycin reduced the levels of IL-1β, IL-6, and TNF, while only the lower concentration of the drug revealed a neuroprotective effect.

Microglia, an immune cell of the central nervous system, plays a pivotal role in neurodegenerative processes and can modulate the production of inflammatory mediators. We thus evaluated whether this compound alters microglia activation in QA-injected mice immediately after the evaluation of the motor performance. We observed that microglia from QA-injected mice were more activated than the PBS-injected controls, as demonstrated by their enlarged cell bodies and retraction of the projections. However, rapamycin did not change the morphology of microglia. This lack of effect might be because we used just one administration, and the morphology of the cells was evaluated only at 2 days after the QA stimulus. Finally, this result, together with the data of the cytokines, suggests that microglia activation and neuroinflammation does not seem to mediate the neuroprotective effects of rapamycin in this model.

Other important cells from the central nervous system that are also involved in neurodegenerative processes are the astrocytes [56]. In the present study, we observed a reduced density of astrocytes in the group treated with QA. Considering the important physiological role of these immune cells in glutamate homeostasis, a reduction in their number would lead to a lower reuptake of this neurotransmitter. Since QA also enhances the release of glutamate, both effects could enhance the excitotoxic process. Therefore, one could speculate that a possible mechanism by which the lower dose of rapamycin partially prevents neurodegeneration and motor impairment would be by avoiding the reduction in astrocyte density induced by QA, which could contribute to maintain their physiological functions in the striatum.

## Conclusions

In conclusion, we demonstrated that mTOR inhibition with rapamycin reduced the motor impairment and neurodegeneration in an animal model of excitotoxicity, providing evidence of the involvement of mTOR in excitotoxic events. Our data suggest that the neuroprotective effects of rapamycin against QA-induced excitotoxicity might be due to different mechanisms including modulation of glutamate release and maintenance of the viability of the astrocytes. However, further studies are necessary to determine the precise mechanism of neuroprotection induced by rapamycin in excitotoxicity. Finally, considering that higher concentrations of rapamycin enhanced the release of glutamate by synaptosomes, a possible neurotoxic effect of the drug should also be investigated in other models.

## Additional files

Additional file 1: Figure S1. (A) Coordinates used for the injection of rapamycin and quinolinic acid (modified from Paxinos, 2001) and (B) scheme of the experimental design.
Additional file 2: Figure S2. Effect of rapamycin on body weight (g) at basal time (0 day) and 2 days after QA administration (*n* = 8 animals/group). Rapamycin was injected 15 min before QA injection into the striatum, and the weigh was checked before the injection of the drugs and after 2 days. Results are expressed as mean ± SEM. No statistical difference was observed (two-way ANOVA followed by Bonferroni test).
Additional file 3: The datasets supporting the conclusions.

## Abbreviations

BDNF: Brain-derived neurotrophic factor
CBA: Cytometric bead array
CEUA: Ethics Committee on Animal Experimentation
EPSC: Excitatory postsynaptic current
FJC: Fluoro-Jade C
GDH: Glutamate dehydrogenase
GFAP: Glial fibrillary acidic protein
HD: Huntington’s disease
Iba-1: Ionized calcium-binding adapter molecule 1
IFN: Interferon
IL: Interleukin
LPS: Lipopolysaccharide
mTOR: Mammalian target of rapamycin
NGF: Nerve growth factor
PFA: Paraformaldehyde
QA: Quinolinic acid
Rapa: Rapamycin
TNF-α: Tumor necrosis factor alpha
