# Supplementary figures and images for: Neuroprotective effects of intrastriatal injection of rapamycin in a mouse model of excitotoxicity induced by quinolinic acid
Source: J Neuroinflammation. 2017 Jan 31;14:25. doi: 10.1186/s12974-017-0793-x (PMC5282622; doi:10.1186/s12974-017-0793-x)

A)

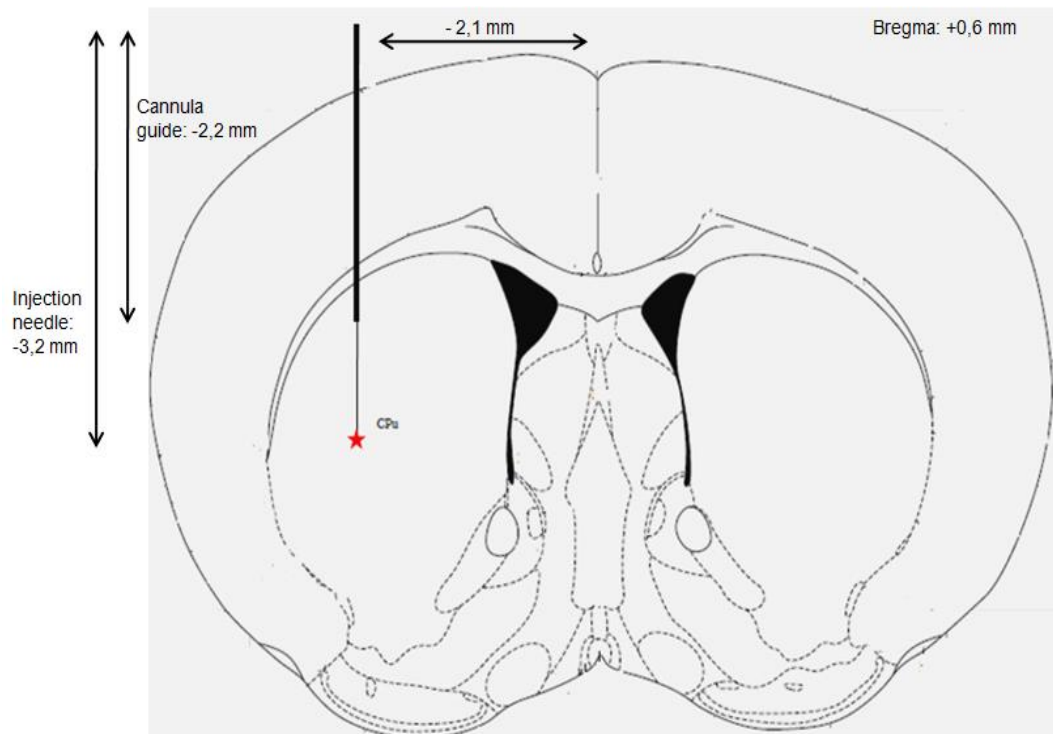

B)

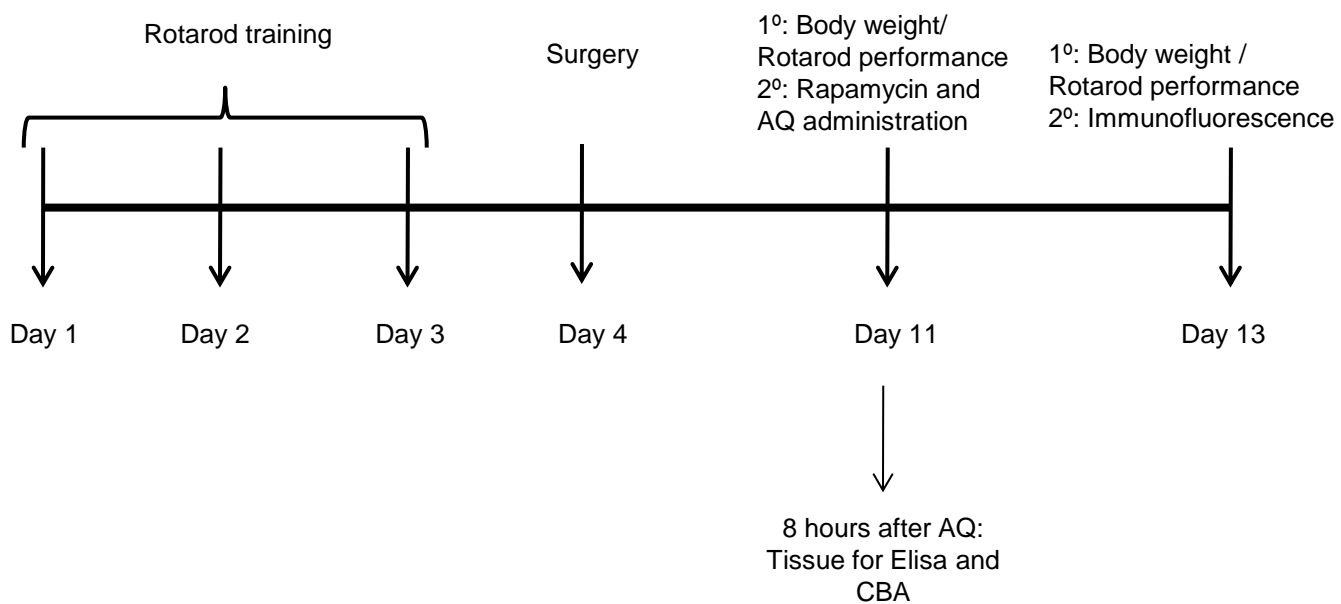

Supplement: Additional file 1: Figure S1. — (A) Coordinates used for the injection of rapamycin and quinolinic acid (modified from Paxinos, 2001) and (B) scheme of the experimental design. [file 12974_2017_793_MOESM1_ESM.pdf]

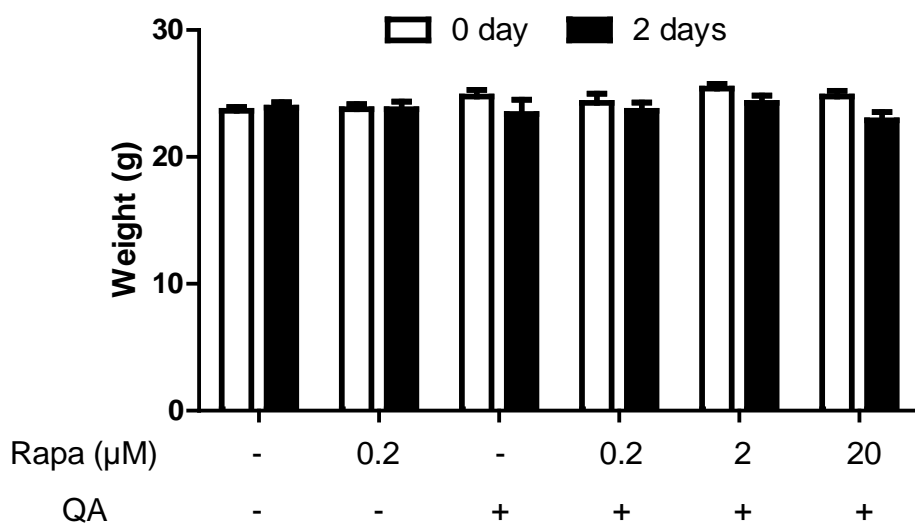

Supplementary Fig. 2

Supplement: Additional file 2: Figure S2. — Effect of rapamycin on body weight (g) at basal time (0 day) and 2 days after QA administration (n = 8 animals/group). Rapamycin was injected 15 min before QA injection into the striatum, and the weigh was checked before the injection of the drugs and after 2 days. Results are expressed as mean ± SEM. No statistical difference was observed (two-way ANOVA followed by Bonferroni test). [file 12974_2017_793_MOESM2_ESM.pdf]
