# Supplementary material for: Neuroprotective effects of intrastriatal injection of rapamycin in a mouse model of excitotoxicity induced by quinolinic acid
Source: J Neuroinflammation. 2017 Jan 31;14:25. doi: 10.1186/s12974-017-0793-x (PMC5282622; doi:10.1186/s12974-017-0793-x)
Supplement: Additional file 3: — The datasets supporting the conclusions. [file 12974_2017_793_MOESM3_ESM.pdf]

SUPPLEMENTARY DATA

Raw data - Figure 1

% seconds as compared with basal

Vehicle + PBS	Rapa 0.2 + PBS	Vehicle + QA 200	Rapa 0.2 + QA 200	Rapa 2 + QA 200	Rapa 20 + QA 200
171,9	86,9	38,6	101,1	55,4	109,3
169,6	139,4	56,2	101,1	106,4	13,3
122,5	124,1	9,1	26,8	3,4	103,8
175,7	52	45,2	102,6	83,4	36,8
88,1	116,1	74,2	64,7	9,4	21,1
99,3	79,8	97,7	172,1	16,9	6,6
36,3	153,5	87,5	16,6	60,2	64,6
97,1	163,6	45	120,1	13,8	174,5
89,1		74,8	120,3	77,9	113,8
96		92,1	149,7	81,3	27,4
74,7		78	63,1	67,7	15,4
98,3		70,5	68,9	31,6	60
123,8		68,7	40,3	48,6	206
101,7			134,2	75,9	111,7
			89.4		

SUPPLEMENTARY DATA

Raw data – Figure 2

cells / mm^2

Vehicle + PBS	Vehicle + QA 200	Rapa 0.2 + QA 200	Rapa 2 + QA 200	Rapa 20 + QA 200
0,00006593	0,00038625	0,00025345	0,00035799	0,00018933
0,00005437	0,00028394	0,00025398	0,00032015	0,00030145
0,00003145	0,0002119	0,00026239	0,0002439	0,00019098
0,00005411	0,00033242	0,00013107	0,00029885	0,0002281
0,00000385	0,00034347	0,00018794	0,00030882	0,00026943
0,00000889		0,00018664	0,00025127	
0,00000432		0,00015253		

SUPPLEMENTARY DATA

Raw data – Figure 3

Glutamate relase (nMol/mg of protein)

KCL	PBS	QA_10 mM	QA_5 mM	QA_0.5 mM	QA_0.1 mM
54,6875	37,04637	258,9799	121,3992	53,125	83,33334
45,53312	47,95259	282,8351	124,6936	62,5	25,58479
26,26568	15,27309	168,4669		86,17424	68,45238

Glutamate release (% of QA group)

KCl	PBS	QA 5mM	Rapa 0.0001nM+ QA 5mM	Rapa 0.001nM+Q A 5mM	Rapa 0.05nM+QA 5mM	Rapa 0.25nM+QA 5mM
0,8438104	2,293592	29,0063	38,24446	92,03006	120,3827	202,79
1,581473	2,526438	147,0392	9,353518	125,2118	159,9353	90,44333
1,991208	11,22537	47,28137	19,9213	79,172	252,8381	183,3908
3,511448	4,339294	176,6731	18,40929	108,9217	136,5946	
2,343265	33,1403	85,36245	62,59767	21,0634		
2,996344	8,900649	108,7583		16,63208		
11,87992	8,655149	105,8792		17,14577		
16,50738	3,065053	135,0118		27,21307		
11,62102	4,734305	50,44202		20,97952		
		114,5461				

SUPPLEMENTARY DATA

Raw data – Figure 4

BDNF (pg/mg of protein)

Vehicle + PBS	Vehicle + QA 200	Rapa 0.2 + QA 200	Rapa 2 + QA 200	Rapa 20 + QA 200
544,976	337,59	389,735	401,024	399,866
692,675	475,153	454,368	289,644	404,792
409,749	443,395	556,819	433,258	378,631
958,755	614,482	384,249	588,918	547,469
725,178	376,826	660,524	652,053	515,813
391,234	572,615	354,14	494,364	501,72
	554,118	675,592	230,818	260,729

NGF (pg/mg of protein)

Vehicle + PBS	Vehicle + QA 200	Rapa 0.2 + QA 200	Rapa 2 + QA 200	Rapa 20 + QA 200
315,527	285,03	269,462	187,642	208,324
473,249	214,727	330,722	136,585	524,633
803,598	532,176	358,875	582,649	100,755
423,464	201,191	192,936	393,165	409,293
280,106	302,038	450,163	449,313	331,593
	253,688	222,414	482,307	153,223
		594,018	101,415	263,993

SUPPLEMENTARY DATA

Raw data – Figure 5

IL-1β (pg/mg of protein)

Vehicle + PBS	Vehicle + QA 200	Rapa 0.2 + QA 200	Rapa 2 + QA 200	Rapa 20 + QA 200
528,156	495,503	458,077	197,555	205,689
467,451	968,268	235,196	176,945	294,805
302,714	467,778	317,032	599,584	313,041
598,292	1069,627	230,019	343,754	176,983
528,159	504,36	255,459	238,631	566,087
388,644	699,872	376,884	128,938	419,173
				193,065

IL-6 (pg/mg of protein)

Vehicle + PBS	Vehicle + QA 200	Rapa 0.2 + QA 200	Rapa 2 + QA 200	Rapa 20 + QA 200
1235,156	7216,393	3329,378	5660,066	402,38
640,756	11104,52	2181,897	2121,525	774,766
155,806	3934,618	1408,739	786,49	1752,694
1539,635	10837,03	2309,943	7724,218	192,369
739,47	4754,805	5553,904	2344,695	3708,84
1025,268	9200,543	2439,528	2688,119	3764,984
	3738,504	1087,298	501,581	1284,905

TNF-a (pg/mg of protein)

Vehicle + PBS	Vehicle + QA 200	Rapa 0.2 + QA 200	Rapa 2 + QA 200	Rapa 20 + QA 200
18,989	167,186	63,679	104,137	8,14
55,281	254,79	40,688	41,45	11,554
13,299	112,621	25,328	22,799	21,627
57,728	617,617	49,472	81,091	5,951
42,254	123,658	178,23	93,028	59,115
36,643	187,291	53,196	23,284	111,412
	65,381	27,463	15,444	24,496

IL-10 (pg/mg of protein)

Vehicle + PBS	Vehicle + QA 200	Rapa 0.2 + QA 200	Rapa 2 + QA 200	Rapa 20 + QA 200
3,687	1,547	54,271	5,837	1,756
25,557	4,123	39,241	8,025	11,614
6,261	54,665	29,629	25,301	5,037
28,084	48,105	34,887	14,527	1,962
3,902	12,936	38,078	14,35	18,33
5,34	21,675	28,088	12,448	35,407
	2,484		0,872	6,711

IFN-γ (pg/mg of protein)

Vehicle + PBS	Vehicle + QA 200	Rapa 0.2 + QA 200	Rapa 2 + QA 200	Rapa 20 + QA 200
3,293	2,383	2,651	2,569	1,461
6,435	3,763	2,981	1,718	1,967
3,406	2,144	2,795	1,765	3,118
3,965	2,866	2,628	2,796	1,694
2,708	1,948	2,513	2,688	2,089
2,605	2,878	1,643	2,107	2,24
	2,251	2,527	1,052	1,257

IL-2 (pg/mg of protein)

Vehicle + PBS	Vehicle + QA 200	Rapa 0.2 + QA 200	Rapa 2 + QA 200	Rapa 20 + QA 200
6,275	4,676	5,07	3,966	3,114
12,074	6,324	5,653	3,163	3,318
6,266	4,696	5,581	3,33	6,201
8,65	5,723	5,679	5,864	3,287
5,246	3,402	5,344	5,793	4,215
4,182	5,821	3,55	4,134	4,566
	4,216	5,662	2,04	2,405

IL- 4 (pg/mg of protein)

Vehicle + PBS	Vehicle + QA 200	Rapa 0.2 + QA 200	Rapa 2 + QA 200	Rapa 20 + QA 200
1,136	0,867	1,08	0,642	0,503
1,144	1,028	0,952	0,626	0,713
1,445	0,801	0,969	0,734	1,09
1,000	0,979	0,916	1,147	0,651
0,743	0,584	0,623	1,103	0,77
	1,078	0,971	0,766	0,757
	0,721		0,404	0,463

IL-17A (pg/mg of protein)

Vehicle + PBS	Vehicle + QA 200	Rapa 0.2 + QA 200	Rapa 2 + QA 200	Rapa 20 + QA 200
2,356	3,208	2,654	2,24	1,073
2,992	2,802	2,489	2,063	1,379
3,367	1,595	2,596	1,22	2,44
1,97	2,93	2,321	2,378	1,284
2,026	1,375	3,383	1,507	2,025
	3,179	1,616	0,825	1,629
	2,518	1,828		0,945

SUPPLEMENTARY DATA

Raw data – Figure 6

cell area / number of cells (mm^2)

Vehicle + PBS	Vehicle + QA 200	Rapa 0.2 + QA 200	Rapa 2 + QA 200	Rapa 20 + QA 200
0,921	1,829	1,209	1,712	1,486
0,843	1,209	1,172	1,39	1,288
1,232	1,371	1,589	1,379	1,197
1,06	1,387	1,436		1,091

SUPPLEMENTARY DATA

Raw data - Supplementary Figure 2

Weight (g)

0 day

Vehicle + PBS	Rapa 0.2 + PBS	Vehicle + AQ 200	Rapa 0.2 + QA 200	Rapa 2 + QA 200	Rapa 20 + QA 200
23	25	22	24	26	23
23	25	24	23	27	25
24	23	25	27	25	26
24	24	24	25	24	27
24	23	26	24	26	25
22	25	27	21	26	24
25	23	25	27	24	24
24	22	25	23	25	24

2 days

Vehicle + PBS	Rapa 0.2 + PBS	Vehicle + AQ 200	Rapa 0.2 + QA 200	Rapa 2 + QA 200	Rapa 20 + QA 200
23	26	17	26	24	24
24	25	21	24	24	19
23	23	25	25	25	23
24	21	23	24	23	25
25	23	26	21	25	22
22	26	27	21	26	22
26	23	23	25	21	24
24	23	25	23	26	24
